# Overview of Ursolic Acid Potential for the Treatment of Metabolic Disorders, Autoimmune Diseases, and Cancers via Nuclear Receptor Pathways

**DOI:** 10.3390/biomedicines11102845

**Published:** 2023-10-19

**Authors:** Sultan F. Kadasah, Mohamed O. Radwan

**Affiliations:** 1 Department of Biology, Faculty of Science, University of Bisha, P.O. Box 551, Bisha 61922, Saudi Arabia; 2 Medicinal and Biological Chemistry Science Farm Joint Research Laboratory, Faculty of Life Sciences, Kumamoto University, Kumamoto 862-0973, Japan

**Keywords:** ursolic acid, nuclear receptors, NASH, metabolic disorders, autoimmune diseases

## Abstract

Nuclear receptors (NRs) form a family of druggable transcription factors that are regulated by ligand binding to orchestrate multifaceted physiological functions, including reproduction, immunity, metabolism, and growth. NRs represent attractive and valid targets for the management and treatment of a vast array of ailments. Pentacyclic triterpenes (PTs) are ubiquitously distributed natural products in medicinal and aromatic plants, of which ursolic acid (UA) is an extensively studied member, due to its diverse bio-pertinent activities against different cancers, inflammation, aging, obesity, diabetes, dyslipidemia, and liver injury. In fact, PTs share a common lipophilic structure that resembles NRs’ endogenous ligands. Herein, we present a review of the literature on UA’s effect on NRs, showcasing the resulting health benefits and potential therapeutic outcomes. De facto, UA exhibited numerous pharmacodynamic effects on PPAR, LXR, FXR, and PXR, resulting in remarkable anti-inflammatory, anti-hyperlipidemic, and hepatoprotective properties, by lowering lipid accumulation in hepatocytes and mitigating non-alcoholic steatohepatitis (NASH) and its subsequent liver fibrosis. Furthermore, UA reversed valproate and rifampicin-induced hepatic lipid accumulation. Additionally, UA showed great promise for the treatment of autoimmune inflammatory diseases such as multiple sclerosis and autoimmune arthritis by antagonizing RORγ. UA exhibited antiproliferative effects against skin, prostate, and breast cancers, partially via PPARα and RORγ pathways. Herein, for the first time, we explore and provide insights into UA bioactivity with respect to NR modulation.

## 1. Introduction

Encoded by 48 genes, nuclear receptors (NRs) are transcription factors that are categorized into seven subfamilies [1]. They include the receptors for steroid hormones, lipophilic vitamins, sterols, and bile acids and are located in the cytoplasm or the nucleus [2]. NRs play a paramount role in orchestrating diverse biological processes, including metabolism, development, growth, inflammation, and reproduction [1,2,3,4,5,6,7]. Disturbance of NR function may lead to a vast array of illnesses; hence, they are deemed attractive targets that can be modulated by small hydrophobic chemical entities [1,2,5,8,9]. Some NRs have well-characterized ligands, which are hydrophobic small molecules [5]. Others are still considered orphan receptors with unknown endogenous or synthetic ligands [2,10,11,12,13]. 

**Figure 1:**
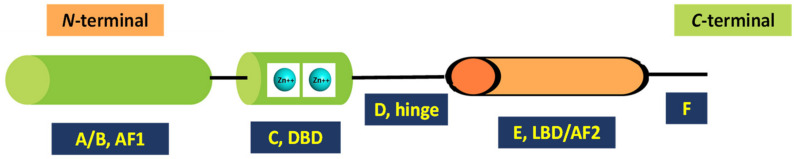
Representation of nuclear receptors’ (NRs) general structure including six regions A–F.

Sequences of NRs share considerable homology and conserved structures, which are divided into six subregions, as shown in Figure 1 [2,14]. The *N*-terminal region involves A/B subregions and has a ligand-independent activation function (AF1). The *N*-terminal is the most divergent among different NRs and is connected to the most conserved C region. The latter represents the DNA binding domain (DBD) that contains two zinc fingers coordinated with cysteines and other basic amino acids. The linker between the C and E region is a flexible, short, and less conservative hinge region, designated as the D region [1,2,14]. The E region encompasses the ligand binding domain (LBD), with a hydrophobic binding site for endogenous ligands and a ligand-dependent activation factor (AF-2). This is followed by the F region, with an unidentified function, towards the variable *C*-terminal [2,15].

Upon ligand binding, conformational changes occur to regulate further transcriptional activity. This happens through recruiting a specific cofactor and binding to a specific DNA-response element (RE) in the corresponding target gene [2]. Imitating the endogenous hydrophobic ligand with a synthetic one that can interact with the LBD is a common approach to modulating NRs’ pharmacological pathways [14,16,17]. The action of ligands is more complicated than it seems, as it occurs in a tissue-specific manner, i.e., the cellular context and type of the recruited cofactor determines the resulting activity. This led to the generation of the term selective modulator of NRs instead of classic agonist–antagonist or inverse agonist terms. 

One of the most explicit examples is the variable pharmacological effect of the widely used estrogen receptor (ERα; NR3A1) selective modulator, raloxifene, for the treatment of breast cancer. Raloxifene antagonizes ERα in breast and uterine tissue, although it is an ERα agonist in bone tissue, which makes it useful for increasing bone density. This differential effect of raloxifene is ascribed to the activation of distinct cofactors in different tissues [2,5]. The concept that a drug can elicit opposing pharmacodynamic activities in different tissues supports the urgent necessity to find a selective modulator of NRs. Finding therapeutically beneficial vitamin D receptors (VDR, NR1I1) modulators without incidence of hypercalcemia is another example. 

NRs may work as homodimers like steroid receptors, including mineralocorticoid receptors (MRs; NR3C2), glucocorticoid receptors (GRs; NR3C1), and the receptors for male and female sex hormones, or may function as heterodimers with an obligatory partner, the retinoic acid X receptor α (RXRα; NR2B1), similar to metabolic NRs [18,19]. Uniquely, retinoic acid receptor-related orphan receptors (RORα; NR1F1, RORβ; NR1F2, and RORγ, NR1F3) can function as monomers or homodimers [20]. 

Many FDA-approved drugs are derived from natural sources, especially in the cancer chemoprevention field [21,22,23]. Pentacyclic triterpenes (PTs) are bioactive plant secondary metabolites with a multitude of bio-pertinent effects [24,25,26,27,28,29,30]. They function to protect plants against pathogens and water loss, thus characterized by their lipophilic scaffold [31]. Researchers have linked them to NR modulation, owing to their structural similarity to the endogenous lipophilic NR modulators [32]. From a chemical perspective, PTs involve mainly four chemical types: oleanane, ursane, lupane, and friedelane, as shown below [24]. 

To date, many natural products have been reported to possess biological activities due to NR modulation [14,15,16,33,34,35,36]. Found in the resin of the guggul plant, guggulsterone is a naturally occurring promiscuous NR modulator with chemoprevention properties [37]. Diterpenoid (−)-acanthoic acid, from the roots of *Rollinia pittieri*, is a potent LXRα agonist with EC_50_ 0.18 μM [38]. The tetracyclic triterpenoid polycarpol, from *Unonopsis glaucopetala*, is a more potent LXRα agonist with EC_50_ 0.03 μM [38]. This may be attributed to the higher similarity of triterpenoids to oxysterols, the endogenous LXRα agonists, than diterpenoids. Theonellasterol, a marine-derived sterol, was identified as an FXR antagonist [39]. 

**Figure 2:**
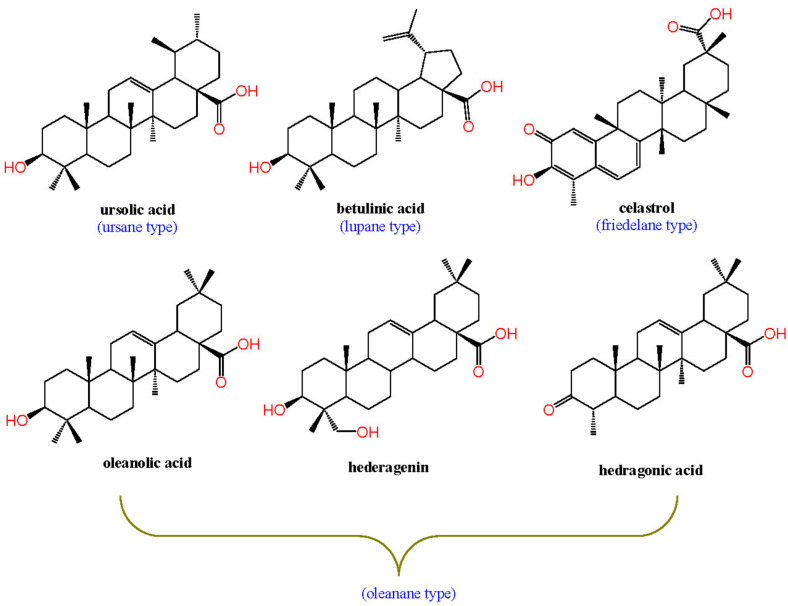
Chemical structures of ursolic acid (UA) and other PTs which modulate NRs, denoting the chemical type of each one.

As anticipated, PTs proved to be prominent NRs ligands, eliciting a multitude of bioactivities due to their high structural similarity to the lipophilic endogenous NRs ligands. A lupane-type PT, betulinic acid, ameliorated non-alcoholic steatohepatitis (NASH) in vivo, via FXR activation [40]. Celastrol, a distinguished friedelane-type PT, is a Nur77 (NR4A1) nuclear receptor with a potential clinical application in Alzheimer’s therapy [41,42]. Notably, oleanolic acid (OA) is one of the most studied oleanane-type PTs with respect to NR modulation, with a multitude of bioactivities against NASH [43,44,45], metabolic disorders [45,46,47], and atherosclerosis [48] via different NR pathways that were recently reviewed [49]. Hedragonic acid, an oleanane-type PT isolated from *Celastrus orbicalatus*, was identified as a hepatoprotective agent against acetaminophen-induced injury through selective FXR agonism. Owing to its high affinity, hedragonic acid was co-crystalized with FXRα LBD (Protein Data Bank ID: 5WZX) [50]. Its analog, hederagenin, promoted FXR mRNA and protein expression with a potential role against colon cancer [51,52]. The chemical structures of UA and other mentioned PTs which modulate NRs were shown in Figure 2.

Ursolic acid (UA), 3-beta-hydroxyurs-12-en-28-oic acid, is one of the most studied ursane-type PTs due to its safety and diverse bioactivities. UA is orally and topically safe in rodents and humans. UA LD_50_ in rodents is quite high: 637 mg/kg for intraperitoneal injection and 8330 mg/kg for oral administration, indicating its high safety margin [53]. UA is abundant in different plant species, including many types of food, medicinal and aromatic plants, especially from the Lamiaceae, Rubiaceae, Araliaceae, Asteraceae, Ericaceae, Saxifragaceae, Verbenaceae, Rosaceae, and Myrtaceae families. Apple and grape skins, marjoram, rosemary, holy basil, and oregano leaves are rich sources of UA [23,54,55]. 

UA modulates different pharmacological pathways, leading to multifaceted health benefits and the prevention of chronic diseases [23,55,56]. In fact, UA demonstrated antiproliferative effects against hepatocellular carcinoma [57,58], lung cancer [59,60], leukemia [61,62,63], breast cancer [64,65], prostate and urogenital cancers [66,67], and cervical cancer [68,69]. Other than its vast anticancer potential, UA was proved to possess ubiquitous biological activities against metabolic diseases, including obesity [70,71,72,73], insulin resistance [74,75,76,77], hyperlipidemia [72,73,78], and atherosclerosis [79], in addition to anti-inflammatory, anti-oxidant, and anti-aging properties, through interfering with different pharmacological pathways, including prominent NR modulation [55,80,81].

**Figure 3:**
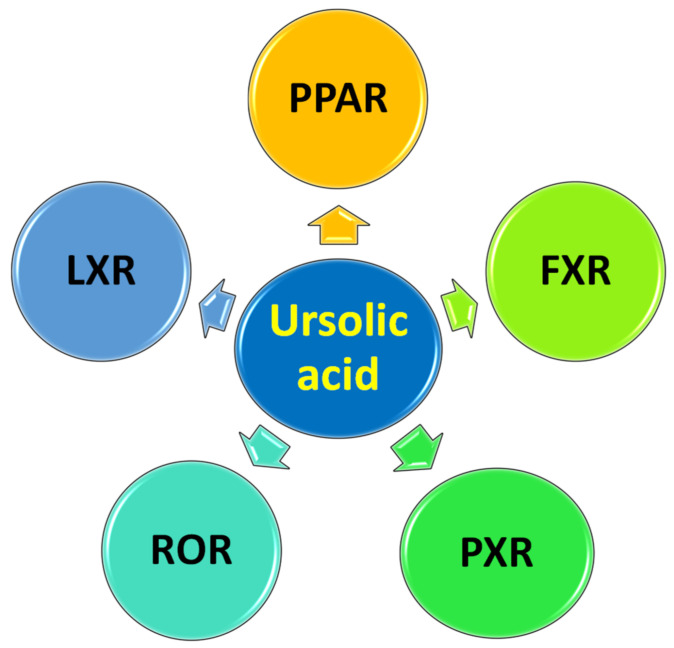
Summary of nuclear receptors (NRs) modulated by ursolic acid (UA).

Provoked by our interest in triterpenes chemistry and bioactivity [29,30,49,82,83] and also in targeting NRs, we systemically compiled all previous studies linking UA to NR modulation. We emphasized the effect of UA on each NR and dissected the resulting bioactivity against metabolic disorders, autoimmune-induced inflammations, and cancers. We focused on only UA as a parent compound, since we did not find any report on its semi-derivatives activity towards NRs. Furthermore, we briefly explained the bioassay experiments used for testing UA. De facto, there are various perspectives on UA highlighting its ubiquitous bioactivities [23,31,56,70,80,84]; however, this is the first one to discuss UA activities from NRs modulation perspective (Figure 3). 

## 2. Methodology

We retrieved the literature from the Web of Science, PubMed, and Google Scholar databases, using the keywords “ursolic acid” and “nuclear receptors” to perform a comprehensive search. This search was performed without publication year limitations, since there was no previously reported review article on the same topic. The outcome was approximately 100 research articles, review articles, and patents, of which 51 were considered for this review. The remaining articles were not extensively investigated as they focused on other types of receptors or other natural products.

## 3. Ursolic Acid Pharmacodynamics towards NRs 

### 3.1. Modulation of Peroxisome-Proliferator-Activated Receptors (PPARs)

PPARs involve three subtypes (PPARα; NR1C1, PPARβ; NR1C2, and PPARγ; NR1C3) that control insulin sensitivity, resistance, and lipid homeostasis, making them valid targets for alleviating metabolic syndrome, hyperlipidemia, and diabetes [85,86,87,88]. PPARα reduces the formation of blood lipids and plays a role in cancer [89], and PPARβ also plays a role in managing blood lipid levels and insulin sensitivity [90,91]. PPARγ controls insulin sensitivity, adipogenesis, neuroprotection [4,92], and inflammation [93]. Fibrates are PPARα modulators used for hyperlipidemia therapy and are represented by fenofibrate and pemafibrate, whereas thiazolidinediones, such as pioglitazone and rosiglitazone, are used for the treatment of diabetes through PPARγ agonism [14,94]. Among different NRs, the UA effect on PPARs is the most explored [95]. 

#### 3.1.1. UA Effect on PPARα

The first report on PT modulation of PPARα and their potential pharmaceutical and cosmeceutical role in dermatology was released in 2005 [96]. Concomitantly, in 2007, Lim et al. showed that topical application of UA to hairless adult mice models enhanced keratinocyte differentiation and led toa subsequent recovery of the epidermal permeability barrier. This effect was clearly observed by examining a biopsy specimen using a light microscope and an electron microscope. The enhanced recovery was hypothesized to be due to PPARα activation. This was validated by immunoblot analysis of PPARα and the keratinocyte differentiation markers involucrin, loricrin, and filaggrin, in human skin keratinocyte cell line HaCaT cells. The test confirmed that UA treatment upregulated the tested protein levels, leading to accelerated recovery. It is worth noting that OA exhibited a similar therapeutic effect [97].

Interestingly, UA’s agonistic effect on PPARα played a pivotal role in its cytotoxic activity against skin cancer through the AMPK pathway. In the mouse squamous carcinoma model, Ca3/7, UA enhanced AMPK phosphorylation at cytotoxic levels, which was reversed by using an AMPK small molecule inhibitor or by AMPK knockdown. As PPARα upregulation has a partial role in skin cancer therapy, the authors investigated this mechanism for UA [89]. Indeed, using the PPARα antagonist GW6471, or the less potent MK886, for one hour prior to adding UA, elevated IC_50_ values of the latter against Ca3/7 or mouse skin papilloma cells MT1/2, as shown by MTT assay. This suggests that the UA cytotoxic effect is partially mediated by PPARα activation [98].

Likewise, Jia et al. confirmed UA-induced activation of PPARα in terms of alleviating hypertriglyceridemia. Having said that, they could not confirm that UA directly binds to PPARα LBD using Biacore surface plasmon resonance (SPR) analysis. However, UA treatment remarkably promoted PPARα mRNA concentration in cultured hepatocytes (HepG2), as shown by qPCR. A luciferase reporter gene assay in the same cells revealed that UA is a PPARα activity upregulator. Furthermore, a 20 µM concentration of UA enhanced PPARα binding to its response element in the responsive genes by 46% and promoted PPARα transactivation consequently. In a dose-dependent manner, UA treatment was significantly proved to have significant hypolipidemic effects by reducing intracellular triglycerides (TGL) and cholesterol accumulation in HepG2 cells. This was accompanied by significant upregulation of the fatty acid transport protein 4 (FATP4) gene in both mRNA and protein levels; FATP4 is a major fatty acid transporter in the liver and a known target gene PPARα. The authors emphasized that UA promotes PPARα transactivation by indirect mechanisms, other than simply binding to its LBD [99].

The same research group moved forward with in vivo validation of their previous in vitro results. They found out that UA can regulate lipid and glucose metabolism in high-fat diet (HFD)-fed mice. UA intake reduced lipid accumulation in adipose tissues and the liver, while increasing muscle mass. Biochemical analysis confirmed that plasma levels of TGL and low- density lipoprotein (LDL) levels were reduced in contrast to high-density lipoprotein (HDL) levels. This was accompanied by improved glucose tolerance and insulin sensitivity. In mice tissue, UA treatment resulted in the over-expression of mRNA and protein levels of PPARα, the activation of its responsive genes that regulate fatty acids uptake and β-oxidation, and the suppression of lipogenic genes [100]. Additionally, UA induced the hepatic expression of the autophagy marker LC3-II, which could partially participate in the hypoglycemic and hypolipidemic role of UA in HFD-fed mice [101].

The anti-hyperlipidemic effect of UA (25 mg/kg) or artesunate (25 mg/kg) alone or in combination (12.5 + 12.5 mg/kg) was assessed in a New Zealand rabbit model on a Western-style diet. UA administration for a couple of months significantly reduced TGL and cholesterol levels in a comparable manner to atorvastatin without a significant effect on LDL level, which was efficiently lowered in the case of UA/artesunate combination. UA alone alleviated hepatocyte steatosis; meanwhile, the combination completely prevented it in the same way as atorvastatin, as displayed by histopathological examination using hematoxylin and eosin (H&E) stains [102]. In liver tissue, UA alone or in combination upregulated mRNA expression of PPARα, which is in agreement with previous studies. 

The potential role of UA in NASH therapy was further investigated by the Li group using the obese NASH Sprague Dawley rat model. UA administration significantly reversed HFD-induced lipid accumulation, NASH, and liver injury and reduced serum ALT, AST, TGL, FFA, and LDL levels in a dose-dependent manner, as revealed by hepatocyte morphologic, histological, and serum biochemical examination. In the same context, UA promoted mRNA and protein levels of PPARα whose knockdown interrupted UA-induced hepatoprotective effect. UA reduced the expression of hepatic inflammatory cytokines, including different interleukins and the tumor necrosis factor α (TNFα). In this model, UA did not affect the activity of PPARγ, farnesoid X receptor (FXR), or liver X receptor (LXR) [103]. Meanwhile, the authors studied the beneficial effect of UA in the human hepatic cell line (HL-7702) model, where it stimulates PPARα mRNA, showing an anti-steatosis effect that was interrupted by PPARα knocking down [103].

Another research group studied the PPARα upregulation effect on alleviating peripheral inflammation and inflammatory hyperalgesia in obese animals. Following the injection of carrageenan into obese Sprague Dawley rats, systemic UA administration mitigated thermal hyperalgesia and paw edema, compared with the control group. At the molecular level, UA lowered the expression of inflammatory mediators, including IL-1β, TNF-α, and NF-κB P65 in the spinal cord of the rats, as shown by the Western blot test. Carrageenan injection into rats’ paws significantly reduced spinal cord PPARα levels in the control HFD groups prior to UA administration, which reversed the process and restored PPARα levels. This means that UA could restore PPARα levels in obese rats’ spinal cords and reduce the expression of inflammatory mediators due to peripheral inflammatory stimulation [104].

UA-induced activation of PPARα is not only beneficial in skin diseases and metabolic disorders, but also in right ventricle hypertrophy (RVH) and remodeling [105]. In a Sprague Dawley monocrotaline-induced RV dysfunction rat model, UA significantly reduced RVH and RV fibrosis, promoted ventricle function, and lowered the increase in cardiomyocyte size and mRNA level of hypertrophic genes and apoptotic cells. From a metabolic aspect, monocrotaline injection remarkably decreased PPARα and PPARγ gene expression; however, UA pretreatment only reversed the abnormal PPARα expression in RV tissue. This opens the way for harnessing UA in the alleviation of RV disorders through the PPARα pathway [106].

#### 3.1.2. UA Effect on PPARγ

The UA effect on PPARγ was described in different aspects of biological activities. PPARγ agonism is well-known to alleviate inflammations in asthmatic animal models [107]. In the BALB/c mice model of allergic bronchial asthma facing methacholine challenge, UA nebulization mitigated methacholine-induced airway hypersensitivity and alleviated airway inflammation. In the ovalbumin-challenged asthma model, ursolic acid (20 mg/kg) reduced eosinophilia bronchoalveolar lavage fluid, neutrophils, and eosinophils, within peripheral blood mononuclear cells (PBMC). Furthermore, it suppressed cytokine, IL-5, IL-13, and IL-17 release, and reduced the level of anti-ovalbumin IgE, in comparison to untreated cells. The authors showed that UA significantly upregulated PPARγ mRNA expression in lung tissue. PPARγ activation was further validated in EL4 T cells and RAW 264.7 macrophages, via qPCR and Western blotting [108].

Wang et al. explored UA neuroprotection effects through the PPARγ pathway in a model of male Sprague Dawley rats with middle cerebral artery occlusion and reperfusion. UA treatment improved the neurological deficit score, promoted the number of intact healthy neurons, and minimized the infarct size compared to the control animals in a dose-dependent manner. This is accompanied by the upregulation of PPARγ, the downregulation of the inflammatory mediators, matrix metalloproteinase-2/9 (MMP2 and MMP9), the increment of the anti-inflammatory factor tissue inhibitor matrix metalloproteinase (1TIMP1), and the interruption of MAPK signaling pathways in brain tissue, as revealed by Western blot and qRT-PCR. Hence, UA can serve as a neuroprotective therapeutic agent, acting via PPARγ agonism, and optimizing the metalloprotease/anti-metalloprotease balance [109].

Another confirmatory report on UA anti-inflammatory activity in the central nervous system (CNS) via PPARγ activation has been recently published. UA enhanced the phenotypic switch of BV2 cells (murine microglia) from M1 polarization, the pro-inflammatory, to M2 polarization, the anti-inflammatory, through the promotion of PPARγ protein expression. Meanwhile, PPARγ activation resulted in the suppression of MMP2 and MMP9 secretion and the increment of 1TIMP1 secretion, which supports the previous results [109]. Notably, those effects were not observed in the case of the co-addition of UA and the potent selective PPARγ antagonist GW9662. In a word, UA protects against neuro-inflammation through the PPARγ pathway, opening the way for its application in ischemic stroke therapy [110]. 

A potential dual role of UA in the treatment of multiple sclerosis (MS) through immunomodulation and neuroregeneration via PPARγ agonism was disclosed by Zhang et al. [111]. In MS mice model using experimental autoimmune encephalomyelitis (EAE), a 25 mg/kg/d of UA reduced CNS inflammation and demyelination; furthermore, in Th1- and Th17-polarizing cultures, UA reduced their differentiation, implying an immunomodulatory effect. At the chronic stage of EAE, UA intake not only hinders further spinal cord myelin damage, but also supports myelin recovery, and protects neurons and axons by promoting oligodendrocyte progenitor cell maturation in CNS lesions. The remyelination-enhancer effect was consistent incuprizone-induced demyelination model in a completely PPARγ-agonistic pathway that disappears incase of PPARγ knockout. In an ex vivo model of lysophosphatidylcholine (LPC)-induced demyelination in organotypic cerebellar slices, UA reduced the expression of inflammatory factors and upregulated anti-inflammatory cytokines and neurotrophins, especially mRNA and the protein level of ciliary neurotrophic factor (CNTF), which, in turn, promoted remyelination. CNTF expression was significantly promoted in astrocytes by UA, and this induction was highly opposed by the PPARγ antagonist GW-9662 [111].

Collectively, UA has a highly promising PPARγ-agonistic character that can be employed for the management of a multitude of diseases, including bronchial asthma, CNS ischemia, and neuro-inflammatory diseases such as MS. Table 1 summarizes the mentioned effects of UA on PPARs and the other NRs in this study and the related bioactivity. 

### 3.2. Modulation of Liver X Receptors (LXRs)

The hydrophobic oxysterols are the endogenous ligands of LXRs, which have two subtypes (LXRα; NR1H3 and LXRβ; NR1H2). Both have shared approximately 70% homology with PPARs. They play a paramount role in lipid and glucose homeostasis, atherosclerosis, and NASH development by regulating hepatic de novo lipogenesis [118,119,120]. Activation of LXRα transactivates hepatic lipogenic genes and LXRα is found to be upregulated in the case of non-alcoholic fatty liver disease (NAFLD); thus, LXR antagonists might be useful for NASH therapy [121,122]. On the contrary, LXRα agonists alleviate the atherosclerotic effect, which is accompanied by severe adverse effects such as hepatic steatosis; this hinders the development of the potent LXRα agonist T090. 

Kuding tea or Ku-Ding-Cha leaves are mainly from *Ilex latifolia* Thunb and *Ilex kudingcha* C.J. Tseng, of the family Aquifoliaceae. This bitter-tasting tea contains high amounts of ursolic acid and has been widely used in China for more than 2000 years as a healthy beverage for the management of obesity, cardiovascular disease, hypertension, and hyperlipidemia [123]. Fan et al. explored the mechanism of action of Kuding tea alcoholic extract [112]. In cell culture, it could interrupt the later stages of 3T3-L1 adipocyte differentiation. Indeed, in the high-fat diet C57BL/6 mice model, the extract reduced weight gain, blood glucose level, and lipid accumulation in hepatocytes. The authors found that the resulting benefits were partially attributed to LXR antagonism. However, they did not figure out which components in the extract were responsible for the activity. 

Later on, Lin et al. identified UA as an LXRα antagonist, in a similar fashion to its oleanane-type analog OA [113,124]. In a dose-dependent manner, UA opposed T090-induced transactivation of LXRα in human hepatocarcinoma cells, as shown by a luciferase reporter assay using an LX response element and SREBP-1c promoter. Consistently, co-treatment with UA attenuated T090-inducedupregulation of LXRα lipogenic target genes, including SREBP-1c, SCD, and FAS. Microscopic examination of Oil Red O stained hepatocytes showed a reduction in TGL and cholesterol accumulation by UA. To validate the present data, the authors went through further in vivo tests using male C57BL/6 mice. Histopathologic examination of the mice liver section, using H&E staining and Oil Red O staining, showed elevated lipid and TG accumulation accompanied by microsteatosis due to T090. This was significantly reversed by the co-administration of UA. In mice hepatocytes, UA showed a similar downregulation effect on lipogenic genesto that in human hepatocytes [113]. 

Molecular docking calculations of UA and T090 into the LXRα ligand binding site (Protein Data Bank ID:1UHL) [125] using the CDOCKER module of Discovery Studio (DS) revealed useful theoretical information on the potential binding pattern. The CDOCKER binding energy of T090, the co-crystalized ligand, was −45.7965 kcal/mol, whereas UA also fitted snugly in the same hydrophobic pocket, with a comparable energy parameter of −37.5211 kcal/mol, reflecting an optimal interaction. UA displayed strong hydrophobic interactions with Phe326, Phe257, Leu331, Trp443, Leu439, Phe254, and Ala261, with a different binding mode from T090. 

UA activity was assessed in human intestinal cells LS174T, and surprisingly, it upregulated ABCA1 and ABCG1 expression instead of the anticipated downregulation. It is worth noting that ABCG1 gene expression decreased upon co-treatment with UA and T090 in HepaRG cells, confirming the cellular-context paradox. This is further confirmed by the lack of UA effect on cellular contents of TG in LS174T cells. It can be deduced that UA suppressed LXRα activation in hepatocytes but not in intestinal cells, suggesting that UA controls LXRα signaling in a cell- and tissue-specific manner due to differential effects on the recruitment of coregulators [113].

The possible clinical application of UA to mitigate the lipogenic side effects of the ani-epileptic drug valproate was tested. De facto, UA significantly opposed valproate induced LXRα transactivation, lipogenic gene expression, and lipid accumulation in HepaRG cells. As we will mention below, UA may also protect against rifampicin-induced hepatic steatosis [114]. 

### 3.3. Modulation of Pregnane X Receptors (PXR) and Constitutive Androstane Receptors (CAR)

Alongside CAR (NR1I3), PXR (NR1I2) is mainly responsible for xenobiotic detoxification by regulating the expression of the metabolic enzyme cytochrome P450 (CYP 450), including the two main types, CYP3A4 and CYP2B6 [126,127]. PXR can be modulated by numerous exogenous and endogenous ligands such as bile acids, steroids, antibiotics like rifampicin, and antimycotics like clotrimazole [128]. Dysregulation of PXR/CAR leads to drug-induced hepatotoxicity, as in the cases of acetaminophen- and isoniazid-induced hepatic injury. 

Using a dual-luciferase reporter gene assay in HepaRG cells, UA, alongside carnosol from *Rosmarinus officinalis*, activated mouse, rat, and human PXR. In terms of human PXR activation, the EC_50_ values of UA and carnosol were 10.77 and 2.22 µM, respectively. UA was confirmed to bind within PXR LBD and activate luciferase activity in cells transfected with a plasmid expressing human PXR LBD. In human colon adenocarcinoma cells, LS180, UA promoted the mRNA expression of the major metabolizing enzyme CYP3A4 and a multi-drug resistance protein 1called ATP binding cassette B1 (ABCB1). In the intestine, this effect enhanced the first-pass metabolism and reduced the oral bioavailability of chemicals metabolized by CYP3A4 and transported by ABCB1 [129].

In 2017, two different reports came out, reporting the promising role of UA and OA in attenuating rifampicin/isoniazid-induced cytotoxicity viamodulation of PXR and its sister NR CAR [114,130]. The presented results were in discrepancy with the above-mentioned outcome of PXR activation. Herein, in human PXR-expressing and CYP3A4 reporter plasmid-transfected HepaRG cells, UA antagonized PXR activity and significantly attenuated the transactivation of the CYP3A4 promoter in a concentration-dependent manner. This inhibitory effect was remarkable incase of co-treatment with the activator rifampicin. Indeed, UA inhibited CYP3A4 mRNA and protein expression. Likewise, UA, through a CAR-dependent mechanism, was involved in the downregulation of the target gene CYP2B6 on both mRNA and protein levels [114]. The catalytic activity of CYP3A4 and CYP2B6 under only UA, or under rifampicin co-treatment, was significantly attenuated. Interestingly, the well-known rifampin-mediated and isoniazid-induced cytotoxicity was reduced by UA co-treatment, as shown by the HepaRG cell viability test. Additionally, UA elevated the intracellular glutathione levels and regeneration capacity in a concentration-dependent manner [114]. 

A supporting claim for the outcome for PXR antagonism by UA was introduced by the same research group in 2018; they evaluated the UA effect on PXR transactivation of lipogenic genes, including S14, SCD, FAS, and FAE. It was revealed that UA could effectively oppose the transient activation of promoters S14 and SCD by rifampicin, as shown by reporter assay. In the presence of rifampicin, UA reduced the mRNA and protein expression of S14, SCD, FAS, and FAE genes. Histopathological examination of stained HepaRG cells by phase-contrast microscope showed rifampicin-induced lipid accumulation and steatosis, which was significantly interrupted by UA [113]. Therefore, UA could serve to lessen the unwanted interactions between transcriptional inducers of CYP450 enzymes and drugs [131].

### 3.4. Modulation of Retinoic Acid Receptor-Related Orphan Receptors (ROR)

As we mentioned above, ROR has three subtypes that possess indispensable roles in immunity, development, and metabolic homeostasis. It is worth noting that the RORγt type is only expressed in immune cells, especially Th17 lymphocytes, where it controls their development and differentiation from CD4^+^ cells [132]. Th17 cells secrete different inflammatory interleukins (ILs), like IL-17 and IL-21, that fight against pathogenic invaders. Nevertheless, its upregulation is linked to autoimmune diseases such as systemic lupus erythematosus, lupus nephritis, psoriasis, rheumatoid arthritis, and MS; thereby, RORγt is a potential target for managing such obstinate diseases [133,134]. Recently, RORγ overexpression was related to the progress of different types of advanced cancers of breast, prostate, and lung [135,136,137]. Endogenous hydroxycholesterols, which have structural similarity to PTs, can bind and modulate RORγt-dependent biological processes [32,138]. 

Indeed, methyl corosolate, uvaol, and OA are three triterpenes found in loquat leaves with in vitro inhibitory effects against RORγt, accompanied by an interruption of Th17 differentiation, with a potential application in lupus nephritis [139]. Furthermore, the titled PTs ameliorated skin inflammation, epidermal hyperplasia, and aberrant keratinocyte proliferation in an imiquimod-induced psoriasis animal model [140]. Digoxin, with its similar structure to hydroxycholesterols, is a well-established RORγt inverse agonist that was co-crystalized with it (Protein Data Bank ID: 3B0W) [141].

The first report claiming that UA acts as a strong and selective inhibitor of RORγt function came out in 2011 by Xu et al. A preliminary high throughput screening of 2000 compounds identified UA as a human and mouse Th17 development and differentiation inhibitor in a dose-dependent manner. At 2 µM, UA inhibited RORγt-mediated, but not RORαt-mediated, IL-17 and IL-17 expression to almost background level in Th17 cells. UA, in a dose-dependent manner, interrupted the binding of RORγt-LBD, but not RORαt-LBD, to its co-activator peptide. Further experiments led to the conclusion that UA exclusively antagonizes RORγt function with IC_50_ of 0.68 µM, while its IC_50_ on Th17 cells was determined to be 0.56 µM. As a result, UA abrogated IL-17 secretion from differentiated Th17 cells of both mouse and human origin. In experimental autoimmune encephalomyelitis mice as an MS model, UA treatment slowed the onset of disease in mice and significantly alleviated the symptoms in comparison with the control group. The CNS of UA-treated mice contained fewer IL-17^+^ and IFN-γ^+^ cells, and their spleen showed less IL-17 production. The study paved the way for UA application in autoimmune disorders and Th17-mediated inflammatory diseases [115].

In addition, UA administration significantly reduced the incidence and severity of collagen-induced autoimmune arthritis in mice models, partially via the inhibition of Th17 differentiation, as shown by flow cytometry. In a dose-dependent manner, mRNA expression of IL-17, IL-21, and RORγt was downregulated in the splenocytes [116]. Owing to its pronounced RORγt antagonism, UA was used as a positive control when testing new inverse agonists [132]. 

A recent interesting report by Zou et al. emphasized the RORγ-dependent anti- proliferative role of UA against triple negative breast cancer (TNBC) cells, HCC70, and prostate cancer (PCa) cell lines C4-2B and 22Rv1 [117]. In the test cell lines, UA lowered RORγ activation, as shown by a luciferase reporter assay, in a dose-dependent manner. In PCa, UA interrupted RORγ-mediated androgen receptors’ (AR) expression and signaling; this was also observed for the variant AR-V7 in C4-2B and 22RV1 cells. The strong anticancer effect of UA was more remarkable in the AR-positive PCa cell line LNCaP but not in the AR-negative PCa cell lines PC3 and DU145. In TNBC, RNA-seq, qRT-PCR, and Western blot analysis showed that UA treatment suppressed the RORγ-mediated mRNA and protein expression of most of the genes controlling cholesterol biosynthesis. Concomitantly, UA disrupted RORγ-controlled apoptosis/cell cycle genes. In conclusion, UA elicits its antiproliferative effect against PCa and TNBC, in part, via targeting RORγ [117].

### 3.5. Modulation of Farnesoid X Receptors FXRs 

FXRs are involved in lipid and bile acid homeostasis, with a significant role in hepatic inflammation and fibrosis, and are widely distributed in organs such as the liver, kidney, intestinal tract, and adrenal gland [142,143,144,145]. Bile acids, the natural ligands of FXR, were identified as potential promoters of colon cancer [37,146]. FXRα; NR1H4 represents a valid target for mitigating primary biliary cirrhosis (PBC), NASH, diabetes [147], and atherosclerosis [6,147,148,149,150]. An FXR modulator, obeticholic acid, was approved for PBC therapy, and further clinical trials are underway to assess it against NASH [151].

A recent report revealed that UA can modulate FXR in rat models with alcoholic liver injury. UA intervention reduced the pathological changes in hepatocytes when examined by hematoxylin–eosin staining. The reduced hepatic steatosis was accompanied by improved cell inflammation and infiltration. In biochemical terms, alanine aminotransferase (ALT), aspartate transaminase (AST), alkaline phosphatase (ALP), and total bile acid (TBA) levels in serum were significantly lessened in comparison to the untreated group. Concomitantly, UA upregulated FXR protein expression and downregulated CYP7A1 and SREBP-1c expression [152]. 

## 4. Conclusions

Selective NR modulation, in the way that introduces health benefits with minimal side effects, is a real challenge due to its conservative structure and unpredictable tissue-specific response. However, finding such a selective modulator can be of huge health benefit in terms of fighting metabolic syndromes that lead to the development of heart and cardiovascular disease. PTs possess a lipophilic structure, making them efficient NR modulators. Indeed, UA demonstrated various pharmacodynamic effects on PPAR, LXR, PXR/CAR, ROR, and FXR. As a result, it exhibited a multitude of health benefits, especially in terms of metabolic disorders, including insulin resistance, diabetes type II, NASH, hyperlipidemia alongside neuroprotection and an anti-asthma effect. Owing to PPARα upregulation, UA can be employed in pharmaceutical and cosmeceutical dermatology and skin cancer. Furthermore, UA showed a comparable anti-hyperlipidemic effect to atorvastatin in vivo. In addition, a significant role of UA in the treatment of autoimmune inflammatory diseases was observed and attributed to RORγt agonism. The latter effect has further conferred anti-proliferative potential to UA in cases of TNBC and PCa. So far, none of the UA semi-synthetic derivatives has been evaluated for NR modulation; therefore, screening of UA derivatives is urgently required, as it may pave the way for finding more potent and selective NR modulators that outperform the parent compound. 

## Figures and Tables

**Table 1:** Summary of ursolic acid (UA) pharmacodynamic effect on nuclear receptors (NRs) and the resulting corresponding therapeutic effect.

Nuclear Receptor Type, UA Pharmacodynamic Effect	Pathology	Type of Study
PPARα (NR1C1), agonist	- Epidermal permeability barrier malfunction	In vivo, hairless adult mice [97]
	- Skin cancer	In vitro, Ca3/7 [98]
	- Hyperlipidemia	In vitro, HepG2 cells [99] and in vivo, HFD fed mice and New Zealand rabbit model on a Western-style diet [101,102]
	- NASH	In vitro, HL-7702 and in vivo, obese NASH Sprague Dawley rats [103]
	- Peripheral inflammation and inflammatory hyperalgesia	In vivo, carrageenan-induced paw edema in obese Sprague Dawley rats [104]
	- Right ventricle hypertrophy (RVH)	In vivo, Sprague Dawley monocrotaline-induced RV dysfunction rats [106]
PPARγ (NR1C3), agonist	- Bronchial asthma	In vivo, BALB/c mice model [108]
	- Neural inflammation and cerebral ischemia	In vitro, BV2 cells [110] and in vivo, male Sprague Dawley rats with middle cerebral artery occlusion and reperfusion [109]
	- Multiple sclerosis (MS)	In vivo, EAE mice and ex vivo, LPC-induced demyelination mice [111]
LXRα (NR1H3), antagonist	- Hepatic lipid accumulation and NASH	In vitro, 3T3-L1 [112,113] and in vivo, C57BL/6 HFD-mice [113]
	- Valproate-induced hepatic steatosis	In vitro, HepaRG cells [113]
PXR (NR1I2)/CAR (NR1I3), antagonist	- Rifampicin-induced hepatic steatosis	In vitro, HepaRG cells [113,114]
RORγ (NR1F3), antagonist/inverse agonist	- Autoimmune encephalitis	In vivo, EAE mice [115]
	- Autoimmune arthritis	In vivo, collagen-induced autoimmune arthritis [116]
	- Breast and prostate cancer	In vitro, HCC70 cells for breast cancer, C4-2B, and 22Rv1 cells for prostate cancer [117]
FXRα (NR1H4), agonist	- NASH	In vivo, rats with alcoholic liver injury

## Data Availability

Not applicable.

## Abbreviations

ABCB1	ATP binding cassette B1
AF	activation function
ALP	alkaline phosphatase
ALT	alanine aminotransferase
AR	androgen receptors
AST	aspartate transaminase
CAR	constitutive androstane receptors
CNS	central nervous system
CNTF	ciliary neurotrophic factor
CYP	cytochrome P
DBD	DNA binding domain
EAE	autoimmune encephalomyelitis
FXR	farnesoid X receptors
HDL	high-density lipoproteins
HFD	high-fat diet
IL	interleukins
LBD	ligand binding domain
LDL	low-density lipoproteins
LPC	lysophosphatidylcholine
LXR	liver X receptors
MMP	matrix metalloproteinase
MS	multiple sclerosis
NASH	non-alcoholic steatohepatitis
NRs	nuclear receptors
OA	oleanolic acid
PBC	primary biliary cirrhosis
PBMC	peripheral blood mononuclear cells
PCa	prostate cancer
PPAR	peroxisome proliferator-activated receptors
PTs	pentacyclic triterpenes
PXR	pregnane X receptors
RE	response element
ROR	retinoic acid receptor-related orphan receptors
RVH	right ventricle hypertrophy
SPR	surface plasmon resonance
TBA	total bile acid
TGL	triglycerides
Th	T helper
TNBC	triple negative breast cancer
TNF	tumor necrosis factor
UA	ursolic acid

## References

[1] Chen T. (2008). Nuclear receptor drug discovery. Curr. Opin. Chem. Biol..

[2] Burris T.P., Solt L.A., Wang Y., Crumbley C., Banerjee S., Griffett K., Lundasen T., Hughes T., Kojetin D.J. (2013). Nuclear receptors and their selective pharmacologic modulators. Pharmacol. Rev..

[3] Mangelsdorf D.J., Thummel C., Beato M., Herrlich P., Schütz G., Umesono K., Blumberg B., Kastner P., Mark M., Chambon P. (1995). The nuclear receptor superfamily: The second decade. Cell.

[4] Cai W., Yang T., Liu H., Han L., Zhang K., Hu X., Zhang X., Yin K.-J., Gao Y., Bennett M.V. (2018). Peroxisome proliferator-activated receptor γ (PPARγ): A master gatekeeper in CNS injury and repair. Prog. Neurobiol..

[5] Frigo D.E., Bondesson M., Williams C. (2021). Nuclear receptors: From molecular mechanisms to therapeutics. Essays Biochem..

[6] Fang Y., Hegazy L., Finck B.N., Elgendy B. (2021). Recent Advances in the Medicinal Chemistry of Farnesoid X Receptor. J. Med. Chem..

[7] Lith S.C., de Vries C.J.M. (2021). Nuclear receptor Nur77: Its role in chronic inflammatory diseases. Essays Biochem..

[8] Penvose A., Keenan J.L., Bray D., Ramlall V., Siggers T. (2019). Comprehensive study of nuclear receptor DNA binding provides a revised framework for understanding receptor specificity. Nat. Commun..

[9] Pan P., Chen X. (2020). Nuclear Receptors as Potential Therapeutic Targets for Myeloid Leukemia. Cells.

[10] Forman B.M., Ruan B., Chen J., Schroepfer G.J., Evans R.M. (1997). The orphan nuclear receptor LXRalpha is positively and negatively regulated by distinct products of mevalonate metabolism. Proc. Natl. Acad. Sci. USA.

[11] Giguère V. (1999). Orphan nuclear receptors: From gene to function. Endocr. Rev..

[12] Kliewer S.A., Lehmann J.M., Willson T.M. (1999). Orphan nuclear receptors: Shifting endocrinology into reverse. Science.

[13] Kumar N., Solt L.A., Conkright J.J., Wang Y., Istrate M.A., Busby S.A., Garcia-Ordonez R.D., Burris T.P., Griffin P.R. (2010). The benzenesulfoamide T0901317 [N-(2,2,2-trifluoroethyl)-N-[4-[2,2,2-trifluoro-1-hydroxy-1-(trifluoromethyl)ethyl]phenyl]-benzenesulfonamide] is a novel retinoic acid receptor-related orphan receptor-alpha/gamma inverse agonist. Mol. Pharmacol..

[14] Yang C., Li Q., Li Y. (2014). Targeting Nuclear Receptors with Marine Natural Products. Mar. Drugs.

[15] Hiebl V., Ladurner A., Latkolik S., Dirsch V.M. (2018). Natural products as modulators of the nuclear receptors and metabolic sensors LXR, FXR and RXR. Biotechnol. Adv..

[16] She J., Gu T., Pang X., Liu Y., Tang L., Zhou X. (2021). Natural Products Targeting Liver X Receptors or Farnesoid X Receptor. Front. Pharmacol..

[17] Krasowski M.D., Ni A., Hagey L.R., Ekins S. (2011). Evolution of promiscuous nuclear hormone receptors: LXR, FXR, VDR, PXR, and CAR. Mol. Cell Endocrinol..

[18] Mangelsdorf D.J., Evans R.M. (1995). The RXR heterodimers and orphan receptors. Cell.

[19] Sever R., Glass C.K. (2013). Signaling by Nuclear Receptors. Cold Spring Harb. Perspect. Biol..

[20] Zhang Y., Luo X., Wu D., Xu Y. (2015). ROR nuclear receptors: Structures, related diseases, and drug discovery. Acta Pharmacol. Sin..

[21] Newman D.J., Cragg G.M. (2016). Natural Products as Sources of New Drugs from 1981 to 2014. J. Nat. Prod..

[22] Koehn F.E., Carter G.T. (2005). The evolving role of natural products in drug discovery. Nat. Rev. Drug Discov..

[23] Seo D.Y., Lee S.R., Heo J.-W., No M.-H., Rhee B.D., Ko K.S., Kwak H.-B., Han J. (2018). Ursolic acid in health and disease. Korean J. Physiol. Pharmacol..

[24] Salvador J.A.R., Leal A.S., Valdeira A.S., Gonçalves B.M.F., Alho D.P.S., Figueiredo S.A.C., Silvestre S.M., Mendes V.I.S. (2017). Oleanane-, ursane-, and quinone methide friedelane-type triterpenoid derivatives: Recent advances in cancer treatment. Eur. J. Med. Chem..

[25] Gutiérrez-Rebolledo G.A., Siordia-Reyes A.G., Meckes-Fischer M., Jiménez-Arellanes A. (2016). Hepatoprotective properties of oleanolic and ursolic acids in antitubercular drug-induced liver damage. Asian Pac. J. Trop. Med..

[26] Ayeleso T.B., Matumba M.G., Mukwevho E. (2017). Oleanolic Acid and Its Derivatives: Biological Activities and Therapeutic Potential in Chronic Diseases. Molecules.

[27] Tsai S.J., Yin M.C. (2008). Antioxidative and anti-inflammatory protection of oleanolic acid and ursolic acid in PC12 cells. J. Food Sci..

[28] Laszczyk M.N. (2009). Pentacyclic triterpenes of the lupane, oleanane and ursane group as tools in cancer therapy. Planta Med..

[29] Radwan M.O., Abd-Alla H.I., Alsaggaf A.T., El-Mezayen H., Abourehab M.A.S., El-Beeh M.E., Tateishi H., Otsuka M., Fujita M. (2023). Gypsogenin Battling for a Front Position in the Pentacyclic Triterpenes Game of Thrones on Anti-Cancer Therapy: A Critical Review—Dedicated to the Memory of Professor Hanaa M. Rady. Molecules.

[30] Radwan M.O., Ismail M.A.H., El-Mekkawy S., Ismail N.S.M., Hanna A.G. (2016). Synthesis and biological activity of new 18β-glycyrrhetinic acid derivatives. Arab. J. Chem..

[31] Gudoityte E., Arandarcikaite O., Mazeikiene I., Bendokas V., Liobikas J. (2021). Ursolic and Oleanolic Acids: Plant Metabolites with Neuroprotective Potential. Int. J. Mol. Sci..

[32] Jin L., Martynowski D., Zheng S., Wada T., Xie W., Li Y. (2010). Structural basis for hydroxycholesterols as natural ligands of orphan nuclear receptor RORgamma. Mol. Endocrinol..

[33] Guo F., Gao Y., Li X., Lei X. (2022). Natural Product 2-Oxokolavenol Is a Novel FXR Agonist. Molecules.

[34] Yamada T., Sugimoto K. (2016). Guggulsterone and Its Role in Chronic Diseases. Adv. Exp. Med. Biol..

[35] Jin L., Feng X., Rong H., Pan Z., Inaba Y., Qiu L., Zheng W., Lin S., Wang R., Wang Z. (2013). The antiparasitic drug ivermectin is a novel FXR ligand that regulates metabolism. Nat. Commun..

[36] Grienke U., Mihály-Bison J., Schuster D., Afonyushkin T., Binder M., Guan S.-H., Cheng C.-R., Wolber G., Stuppner H., Guo D.-A. (2011). Pharmacophore-based discovery of FXR-agonists. Part II: Identification of bioactive triterpenes from Ganoderma lucidum. Bioorg. Med. Chem..

[37] Shishodia S., Azu N., Rosenzweig J.A., Jackson D.A. (2016). Guggulsterone for Chemoprevention of Cancer. Curr. Pharm. Des..

[38] Jayasuriya H., Herath K.B., Ondeyka J.G., Guan Z., Borris R.P., Tiwari S., de Jong W., Chavez F., Moss J., Stevenson D.W. (2005). Diterpenoid, steroid, and triterpenoid agonists of liver X receptors from diversified terrestrial plants and marine sources. J. Nat. Prod..

[39] Renga B., Mencarelli A., D’Amore C., Cipriani S., D’Auria M.V., Sepe V., Chini M.G., Monti M.C., Bifulco G., Zampella A. (2012). Discovery that theonellasterol a marine sponge sterol is a highly selective FXR antagonist that protects against liver injury in cholestasis. PLoS ONE.

[40] Gu M., Zhao P., Zhang S., Fan S., Yang L., Tong Q., Ji G., Huang C. (2019). Betulinic acid alleviates endoplasmic reticulum stress-mediated nonalcoholic fatty liver disease through activation of farnesoid X receptors in mice. Br. J. Pharmacol..

[41] Hu M., Luo Q., Alitongbieke G., Chong S., Xu C., Xie L., Chen X., Zhang D., Zhou Y., Wang Z. (2017). Celastrol-Induced Nur77 Interaction with TRAF2 Alleviates Inflammation by Promoting Mitochondrial Ubiquitination and Autophagy. Mol. Cell.

[42] Liu L., Ma D., Zhuo L., Pang X., You J., Feng J. (2021). Progress and Promise of Nur77-based Therapeutics for Central Nervous System Disorders. Curr. Neuropharmacol..

[43] Liu J., Liu J., Meng C., Gu Q., Huang C., Liu F., Xia C. (2023). NRF2 and FXR dual signaling pathways cooperatively regulate the effects of oleanolic acid on cholestatic liver injury. Phytomedicine.

[44] Liu J., Liu J., Meng C., Huang C., Liu F., Xia C. (2022). Oleanolic acid alleviates ANIT-induced cholestatic liver injury by activating Fxr and Nrf2 pathways to ameliorate disordered bile acids homeostasis. Phytomedicine.

[45] Ma H., Bao Y., Niu S., Wang S., Li Y., He H., Zhang N., Fang W. (2023). Structure Optimization of 12β-O-γ-Glutamyl Oleanolic Acid Derivatives Resulting in Potent FXR Antagonist/Modulator for NASH Therapy. Pharmaceuticals.

[46] Wang Y., Porter W.W., Suh N., Honda T., Gribble G.W., Leesnitzer L.M., Plunket K.D., Mangelsdorf D.J., Blanchard S.G., Willson T.M. (2000). A synthetic triterpenoid, 2-cyano-3,12-dioxooleana-1,9-dien-28-oic acid (CDDO), is a ligand for the peroxisome proliferator-activated receptor gamma. Mol. Endocrinol..

[47] Christensen K.B., Jørgensen M., Kotowska D., Petersen R.K., Kristiansen K., Christensen L.P. (2010). Activation of the nuclear receptor PPARγ by metabolites isolated from sage (*Salvia officinalis* L.). J. Ethnopharmacol..

[48] Pan Y., Zhou F., Song Z., Huang H., Chen Y., Shen Y., Jia Y., Chen J. (2018). Oleanolic acid protects against pathogenesis of atherosclerosis, possibly via FXR-mediated angiotensin (Ang)-(1–7) upregulation. Biomed. Pharmacother..

[49] Radwan M.O., Kadasah S.F., Aljubiri S.M., Alrefaei A.F., El-Maghrabey M.H., El Hamd M.A., Tateishi H., Otsuka M., Fujita M. (2023). Harnessing Oleanolic Acid and Its Derivatives as Modulators of Metabolic Nuclear Receptors. Biomolecules.

[50] Lu Y., Zheng W., Lin S., Guo F., Zhu Y., Wei Y., Liu X., Jin S., Jin L., Li Y. (2018). Identification of an Oleanane-Type Triterpene Hedragonic Acid as a Novel Farnesoid X Receptor Ligand with Liver Protective Effects and Anti-inflammatory Activity. Mol. Pharmacol..

[51] Fallon C.M., Smyth J.S., Quach A., Lajczak-McGinley N., O’toole A., Barrett K.E., Sheridan H., Keely S.J. (2022). Pentacyclic triterpenes modulate farnesoid X receptor expression in colonic epithelial cells: Implications for colonic secretory function. J. Biol. Chem..

[52] Modica S., Murzilli S., Salvatore L., Schmidt D.R., Moschetta A. (2008). Nuclear Bile Acid Receptor FXR Protects against Intestinal Tumorigenesis. Cancer Res..

[53] Lee A.-W., Chen T.-L., Shih C.-M., Huang C.-Y., Tsao N.-W., Chang N.-C., Chen Y.-H., Fong T.-H., Lin F.-Y. (2010). Ursolic Acid Induces Allograft Inflammatory Factor-1 Expression via a Nitric Oxide-Related Mechanism and Increases Neovascularization. J. Agric. Food Chem..

[54] Zhang F., Daimaru E., Ohnishi M., Kinoshita M., Tokuji Y. (2013). Oleanolic Acid and Ursolic Acid in Commercial Dried Fruits. Food Sci. Technol. Res..

[55] Habtemariam S. (2019). Antioxidant and Anti-inflammatory Mechanisms of Neuroprotection by Ursolic Acid: Addressing Brain Injury, Cerebral Ischemia, Cognition Deficit, Anxiety, and Depression. Oxid. Med. Cell. Longev..

[56] Hussain H., Green I.R., Ali I., Khan I.A., Ali Z., Al-Sadi A.M., Ahmed I. (2017). Ursolic acid derivatives for pharmaceutical use: A patent review (2012–2016). Expert Opin. Ther. Pat..

[57] Yan S., Huang C., Wu S., Yin M. (2010). Oleanolic acid and ursolic acid induce apoptosis in four human liver cancer cell lines. Toxicol. In Vitro.

[58] Son H.-S., Kwon H.Y., Sohn E.J., Lee J.-H., Woo H.-J., Yun M., Kim S.-H., Kim Y.-C. (2013). Activation of AMP-activated protein kinase and phosphorylation of glycogen synthase kinase3 β mediate ursolic acid induced apoptosis in HepG2 liver cancer cells. Phytother. Res..

[59] Castrejón-Jiménez N.S., Leyva-Paredes K., Baltierra-Uribe S.L., Castillo-Cruz J., Campillo-Navarro M., Hernández-Pérez A.D., Luna-Angulo A.B., Chacón-Salinas R., Coral-Vázquez R.M., Estrada-García I. (2019). Ursolic and Oleanolic Acids Induce Mitophagy in A549 Human Lung Cancer Cells. Molecules.

[60] Kornel A., Nadile M., Tsiani E. (2022). Evidence of the Beneficial Effects of Ursolic Acid against Lung Cancer. Molecules.

[61] Gao N., Cheng S., Budhraja A., Gao Z., Chen J., Liu E.-H., Huang C., Chen D., Yang Z., Liu Q. (2012). Ursolic acid induces apoptosis in human leukaemia cells and exhibits anti-leukaemic activity in nude mice through the PKB pathway. Br. J. Pharmacol..

[62] Wu B., Wang X., Chi Z.-F., Hu R., Zhang R., Yang W., Liu Z.-G. (2012). Ursolic acid-induced apoptosis in K562 cells involving upregulation of PTEN gene expression and inactivation of the PI3K/Akt pathway. Arch. Pharm. Res..

[63] Ciftci H.I., Radwan M.O., Ozturk S.E., Ulusoy N.G., Sozer E., Ellakwa D.E., Ocak Z., Can M., Ali T.F., Abd-Alla H.I. (2019). Design, Synthesis and Biological Evaluation of Pentacyclic Triterpene Derivatives: Optimization of Anti-ABL Kinase Activity. Molecules.

[64] Wang S., Chang X., Zhang J., Li J., Wang N., Yang B., Pan B., Zheng Y., Wang X., Ou H. (2021). Ursolic Acid Inhibits Breast Cancer Metastasis by Suppressing Glycolytic Metabolism via Activating SP1/Caveolin-1 Signaling. Front. Oncol..

[65] Luo J., Hu Y.-L., Wang H. (2017). Ursolic acid inhibits breast cancer growth by inhibiting proliferation, inducing autophagy and apoptosis, and suppressing inflammatory responses via the PI3K/AKT and NF-κB signaling pathways in vitro. Exp. Ther. Med..

[66] Mu D., Zhou G., Li J., Su B., Guo H. (2018). Ursolic acid activates the apoptosis of prostate cancer via ROCK/PTEN mediated mitochondrial translocation of cofilin-1. Oncol. Lett..

[67] Kornel A., Nadile M., Retsidou M.I., Sakellakis M., Gioti K., Beloukas A., Sze N.S.K., Klentrou P., Tsiani E. (2023). Ursolic Acid against Prostate and Urogenital Cancers: A Review of In Vitro and In Vivo Studies. Int. J. Mol. Sci..

[68] Xu H., Zhou Z., Dong J., Lei M. (2018). Suppression of cervical cancer cell survival by ursolic acid extracted from Catalpa bungei leaves. Pharmacogn. Mag..

[69] Wang S., Meng X., Dong Y. (2017). Ursolic acid nanoparticles inhibit cervical cancer growth in vitro and in vivo via apoptosis induction. Int. J. Oncol..

[70] Mancha-Ramirez A.M., Slaga T.J. (2016). Ursolic Acid and Chronic Disease: An Overview of UA’s Effects On Prevention and Treatment of Obesity and Cancer. Adv. Exp. Med. Biol..

[71] Tian C., Li J., Bao Y., Gao L., Song L., Li K., Sun M. (2023). Ursolic acid ameliorates obesity of mice fed with high-fat diet via alteration of gut microbiota and amino acid metabolism. Front. Microbiol..

[72] Rao V.S., de Melo C.L., Queiroz M.G.R., Lemos T.L., Menezes D.B., Melo T.S., Santos F.A., Silva F.S.G., Oliveira P.J., Duarte M.F. (2011). Ursolic acid, a pentacyclic triterpene from Sambucus australis, prevents abdominal adiposity in mice fed a high-fat diet. J. Med. Food.

[73] Kim J., Jang D.S., Kim H., Kim J.S. (2009). Anti-lipase and lipolytic activities of ursolic acid isolated from the roots of Actinidia arguta. Arch. Pharm. Res..

[74] Kunkel S.D., Elmore C.J., Bongers K.S., Ebert S.M., Fox D.K., Dyle M.C., Bullard S.A., Adams C.M. (2012). Ursolic acid increases skeletal muscle and brown fat and decreases diet-induced obesity, glucose intolerance and fatty liver disease. PLoS ONE.

[75] Katashima C.K., Silva V.R., Gomes T.L., Pichard C., Pimentel G.D. (2017). Ursolic acid and mechanisms of actions on adipose and muscle tissue: A systematic review. Obes. Rev..

[76] Jung S.H., Ha Y.J., Shim E.K., Choi S.Y., Jin J.L., Yun-Choi H.S., Lee J.R. (2007). Insulin-mimetic and insulin-sensitizing activities of a pentacyclic triterpenoid insulin receptor activator. Biochem. J..

[77] Silva F.S.G., Oliveira P.J., Duarte M.F. (2016). Oleanolic, Ursolic, and Betulinic Acids as Food Supplements or Pharmaceutical Agents for Type 2 Diabetes: Promise or Illusion?. J. Agric. Food Chem..

[78] Azevedo M.F., Camsari C., Sá C.M., Lima C.F., Fernandes-Ferreira M., Pereira-Wilson C. (2010). Ursolic acid and luteolin-7-glucoside improve lipid profiles and increase liver glycogen content through glycogen synthase kinase-3. Phytother. Res..

[79] Pozo M., Castilla V., Gutierrez C., de Nicolás R., Egido J., González-Cabrero J. (2006). Ursolic acid inhibits neointima formation in the rat carotid artery injury model. Atherosclerosis.

[80] Kashyap D., Sharma A., Tuli H.S., Punia S., Sharma A.K. (2016). Ursolic Acid and Oleanolic Acid: Pentacyclic Terpenoids with Promising Anti-Inflammatory Activities. Recent Pat. Inflamm. Allergy Drug Discov..

[81] Bakhtiari N., Moslemee-Jalalvand E., Kazemi J. (2017). Ursolic acid: A versatile triterpenoid compound in regulating the aging. Physiol. Pharmacol..

[82] Ciftci H.I., Ozturk S.E., Ali T.F.S., Radwan M.O., Tateishi H., Koga R., Ocak Z., Can M., Otsuka M., Fujita M. (2018). The First Pentacyclic Triterpenoid Gypsogenin Derivative Exhibiting Anti-ABL1 Kinase and Anti-chronic Myelogenous Leukemia Activities. Biol. Pharm. Bull..

[83] Ciftci H.I., Radwan M.O., Sever B., Hamdy A.K., Emirdağ S., Ulusoy N.G., Sozer E., Can M., Yayli N., Araki N. (2021). EGFR-Targeted Pentacyclic Triterpene Analogues for Glioma Therapy. Int. J. Mol. Sci..

[84] Nguyen H.N., Ullevig S.L., Short J.D., Wang L., Ahn Y.J., Asmis R. (2021). Ursolic Acid and Related Analogues: Triterpenoids with Broad Health Benefits. Antioxidants.

[85] Berger J., Moller D.E. (2002). The mechanisms of action of PPARs. Annu. Rev. Med..

[86] Zhang F., Lavan B.E., Gregoire F.M. (2007). Selective Modulators of PPAR-gamma Activity: Molecular Aspects Related to Obesity and Side-Effects. PPAR Res..

[87] Allen T., Zhang F., Moodie S.A., Clemens L.E., Smith A., Gregoire F., Bell A., Muscat G.E., Gustafson T.A. (2006). Halofenate is a selective peroxisome proliferator-activated receptor gamma modulator with antidiabetic activity. Diabetes.

[88] Zheng W., Feng X., Qiu L., Pan Z., Wang R., Lin S., Hou D., Jin L., Li Y. (2013). Identification of the antibiotic ionomycin as an unexpected peroxisome proliferator-activated receptor γ (PPARγ) ligand with a unique binding mode and effective glucose-lowering activity in a mouse model of diabetes. Diabetologia.

[89] Thuillier P., Anchiraico G.J., Nickel K.P., Maldve R.E., Gimenez-Conti I., Muga S.J., Liu K.-L., Fischer S.M., Belury M.A. (2000). Activators of peroxisome proliferator-activated receptor-alpha partially inhibit mouse skin tumor promotion. Mol. Carcinog..

[90] Leone T.C., Weinheimer C.J., Kelly D.P. (1999). A critical role for the peroxisome proliferator-activated receptor alpha (PPARalpha) in the cellular fasting response: The PPARalpha-null mouse as a model of fatty acid oxidation disorders. Proc. Natl. Acad. Sci. USA.

[91] Sanderson L.M., Degenhardt T., Koppen A., Kalkhoven E., Desvergne B., Müller M., Kersten S. (2009). Peroxisome proliferator-activated receptor beta/delta (PPARbeta/delta) but not PPARalpha serves as a plasma free fatty acid sensor in liver. Mol. Cell. Biol..

[92] Hou G., Yin Y., Han D., Wang Q.-Y., Kang J. (2015). Rosiglitazone attenuates the metalloprotease/anti-metalloprotease imbalance in emphysema induced by cigarette smoke: Involvement of extracellular signal-regulated kinase and NFκB signaling. Int. J. Chron Obs. Pulmon Dis..

[93] Fajas L., Auboeuf D., Raspé E., Schoonjans K., Lefebvre A.-M., Saladin R., Najib J., Laville M., Fruchart J.-C., Deeb S. (1997). The Organization, Promoter Analysis, and Expression of the Human PPARγ Gene*. J. Biol. Chem..

[94] Orasanu G., Ziouzenkova O., Devchand P.R., Nehra V., Hamdy O., Horton E.S., Plutzky J. (2008). The PPARγ Agonist Pioglitazone Represses Inflammation In A PPARα-Dependent Manner In Vitro and In Vivo In Mice. J. Am. Coll. Cardiol..

[95] Enayati A., Ghojoghnejad M., Roufogalis B.D., Maollem S.A., Sahebkar A. (2022). Impact of Phytochemicals on PPAR Receptors: Implications for Disease Treatments. PPAR Res..

[96] Lee H.K., Nam G.W., Kim S.H., Lee S.H. (2006). Phytocomponents of triterpenoids, oleanolic acid and ursolic acid, regulated differently the processing of epidermal keratinocytes via PPAR-α pathway. Exp. Dermatol..

[97] Lim S.W., Hong S.P., Jeong S.W., Kim B., Bak H., Ryoo H.C., Lee S.H., Ahn S.K. (2007). Simultaneous effect of ursolic acid and oleanolic acid on epidermal permeability barrier function and epidermal keratinocyte differentiation via peroxisome proliferator-activated receptor-α. J. Dermatol..

[98] Junco J.J., Cho J., Mancha A., Malik G., Wei S.-J., Kim D.J., Liang H., DiGiovanni J., Slaga T.J. (2018). Role of AMPK and PPARα in the anti-skin cancer effects of ursolic acid. Mol. Carcinog..

[99] Jia Y., Bhuiyan M.J.H., Jun H.-J., Lee J.H., Hoang M.H., Lee H.-J., Kim N., Lee D., Hwang K.Y., Hwang B.Y. (2011). Ursolic acid is a PPAR-α agonist that regulates hepatic lipid metabolism. Bioorg. Med. Chem. Lett..

[100] Kimura S., Noda T., Yoshimori T. (2007). Dissection of the autophagosome maturation process by a novel reporter protein, tandem fluorescent-tagged LC3. Autophagy.

[101] Jia Y., Kim S., Kim J., Kim B., Wu C., Lee J.H., Jun H.-J., Kim N., Lee D., Lee S.-J. (2015). Ursolic acid improves lipid and glucose metabolism in high-fat-fed C57BL/6J mice by activating peroxisome proliferator-activated receptor alpha and hepatic autophagy. Mol. Nutr. Food Res..

[102] Wang Y.L., Wang Z.J., Shen H.L., Yin M., Tang K.X. (2013). Effects of artesunate and ursolic acid on hyperlipidemia and its complications in rabbit. Eur. J. Pharm. Sci..

[103] Li S., Meng F., Liao X., Wang Y., Sun Z., Guo F., Li X., Meng M., Li Y., Sun C. (2014). Therapeutic role of ursolic acid on ameliorating hepatic steatosis and improving metabolic disorders in high-fat diet-induced non-alcoholic fatty liver disease rats. PLoS ONE.

[104] Zhang Y., Song C., Li H., Hou J., Li D. (2016). Ursolic acid prevents augmented peripheral inflammation and inflammatory hyperalgesia in high-fat diet-induced obese rats by restoring downregulated spinal PPARα. Mol. Med. Rep..

[105] Xu Y., Gu Q., Liu N., Yan Y., Yang X., Hao Y., Qu C. (2017). PPARγ Alleviates Right Ventricular Failure Secondary to Pulmonary Arterial Hypertension in Rats. Int. Heart J..

[106] Gao X., Zhang Z., Li X.B., Wei Q., Li H., Li C., Chen H., Liu C., He K. (2020). Ursolic Acid Improves Monocrotaline-Induced Right Ventricular Remodeling by Regulating Metabolism. J. Cardiovasc. Pharmacol..

[107] Hirasawa H., Chiba T., Ueki S., Kamada Y., Ito W., Takeda M., Fujita M., Kato H., Kayaba H., Chihara J. (2008). The synthetic PPARgamma agonist troglitazone inhibits eotaxin-enhanced eosinophil adhesion to ICAM-1-coated plates. Int. Arch. Allergy Immunol..

[108] Kim S.-H., Hong J.-H., Lee Y.-C. (2013). Ursolic acid, a potential PPARγ agonist, suppresses ovalbumin-induced airway inflammation and Penh by down-regulating IL-5, IL-13, and IL-17 in a mouse model of allergic asthma. Eur. J. Pharmacol..

[109] Wang Y., He Z., Deng S. (2016). Ursolic acid reduces the metalloprotease/anti-metalloprotease imbalance in cerebral ischemia and reperfusion injury. Drug Des. Devel. Ther..

[110] Wang Y., Qiu L., Deng S., Liu F., He Z., Li M., Wang Y. (2023). Ursolic acid promotes microglial polarization toward the M2 phenotype via PPARγ regulation of MMP2 transcription. Neurotoxicology.

[111] Zhang Y., Li X., Ciric B., Curtis M.T., Chen W.-J., Rostami A., Zhang G.-X. (2020). A dual effect of ursolic acid to the treatment of multiple sclerosis through both immunomodulation and direct remyelination. Proc. Natl. Acad. Sci. USA.

[112] Fan S., Zhang Y., Hu N., Sun Q., Ding X., Li G., Zheng B., Gu M., Huang F., Sun Y.-Q. (2012). Extract of Kuding tea prevents high-fat diet-induced metabolic disorders in C57BL/6 mice via liver X receptor (LXR) β antagonism. PLoS ONE.

[113] Lin Y.-N., Wang C.C.N., Chang H.-Y., Chu F.-Y., Hsu Y.-A., Cheng W.-K., Ma W.-C., Chen C.-J., Wan L., Lim Y.-P. (2018). Ursolic Acid, a Novel Liver X Receptor α (LXRα) Antagonist Inhibiting Ligand-Induced Nonalcoholic Fatty Liver and Drug-Induced Lipogenesis. J. Agric. Food Chem..

[114] Chang H.-Y., Chen C.-J., Ma W.-C., Cheng W.-K., Lin Y.-N., Lee Y.-R., Chen J.-J., Lim Y.-P. (2017). Modulation of pregnane X receptor (PXR) and constitutive androstane receptor (CAR) activation by ursolic acid (UA) attenuates rifampin-isoniazid cytotoxicity. Phytomedicine.

[115] Xu T., Wang X., Zhong B., Nurieva R.I., Ding S., Dong C. (2011). Ursolic Acid Suppresses Interleukin-17 (IL-17) Production by Selectively Antagonizing the Function of RORγt Protein*. J. Biol. Chem..

[116] Baek S.-Y., Lee J., Lee D.-G., Park M.-K., Lee J., Kwok S.-K., Cho M.-L., Park S.-H. (2014). Ursolic acid ameliorates autoimmune arthritis via suppression of Th17 and B cell differentiation. Acta Pharmacol. Sin..

[117] Zou H., Yang Y., Chen H.-W. (2023). Natural compounds ursolic acid and digoxin exhibit inhibitory activities to cancer cells in RORγ-dependent and -independent manner. Front. Pharmacol..

[118] Dixon E.D., Nardo A.D., Claudel T., Trauner M. (2021). The Role of Lipid Sensing Nuclear Receptors (PPARs and LXR) and Metabolic Lipases in Obesity, Diabetes and NAFLD. Genes.

[119] Lin Z., Zhang Y., Zhang Y., Shen H., Hu L., Jiang H., Shen X. (2008). Oleanolic acid derivative NPLC441 potently stimulates glucose transport in 3T3-L1 adipocytes via a multi-target mechanism. Biochem. Pharmacol..

[120] Edwards P.A., Kennedy M.A., Mak P.A. (2002). LXRs; oxysterol-activated nuclear receptors that regulate genes controlling lipid homeostasis. Vasc. Pharmacol..

[121] Higuchi N., Kato M., Shundo Y., Tajiri H., Tanaka M., Yamashita N., Kohjima M., Kotoh K., Nakamuta M., Takayanagi R. (2008). Liver X receptor in cooperation with SREBP-1c is a major lipid synthesis regulator in nonalcoholic fatty liver disease. Hepatol. Res..

[122] Griffett K., Burris T.P. (2023). Development of LXR inverse agonists to treat MAFLD, NASH, and other metabolic diseases. Front. Med..

[123] Wüpper S., Lüersen K., Rimbach G. (2020). Chemical Composition, Bioactivity and Safety Aspects of Kuding Tea—From Beverage to Herbal Extract. Nutrients.

[124] Lin Y.-N., Chang H.-Y., Wang C.C.N., Chu F.-Y., Shen H.-Y., Chen C.-J., Lim Y.-P. (2018). Oleanolic Acid Inhibits Liver X Receptor Alpha and Pregnane X Receptor to Attenuate Ligand-Induced Lipogenesis. J. Agric. Food Chem..

[125] Svensson S., Östberg T., Jacobsson M., Norström C., Stefansson K., Hallén D., Johansson I.C., Zachrisson K., Ogg D., Jendeberg L. (2003). Crystal structure of the heterodimeric complex of LXRα and RXRβ ligand-binding domains in a fully agonistic conformation. EMBO J..

[126] Wada T., Gao J., Xie W. (2009). PXR and CAR in energy metabolism. Trends Endocrinol. Metab..

[127] Kliewer S.A., Goodwin B., Willson T.M. (2002). The nuclear pregnane X receptor: A key regulator of xenobiotic metabolism. Endocr. Rev..

[128] Ekins S., Chang C., Mani S., Krasowski M.D., Reschly E.J., Iyer M., Kholodovych V., Ai N., Welsh W.J., Sinz M. (2007). Human pregnane X receptor antagonists and agonists define molecular requirements for different binding sites. Mol. Pharmacol..

[129] Seow C.L., Lau A.J. (2017). Differential activation of pregnane X receptor by carnosic acid, carnosol, ursolic acid, and rosmarinic acid. Pharmacol. Res..

[130] Lin Y.-N., Chen C.-J., Chang H.-Y., Cheng W.-K., Lee Y.-R., Chen J.-J., Lim Y.-P. (2017). Oleanolic Acid-Mediated Inhibition of Pregnane X Receptor and Constitutive Androstane Receptor Attenuates Rifampin-Isoniazid Cytotoxicity. J. Agric. Food Chem..

[131] Zhang Z.-H., Tang J.-H., Zhan Z.-L., Zhang X.-L., Wu H.-H., Hou Y.-N. (2012). Cellular toxicity of isoniazid together with rifampicin and the metabolites of isoniazid on QSG-7701 hepatocytes. Asian Pac. J. Trop. Med..

[132] Prà M.D., Carta D., Szabadkai G., Suman M., Frión-Herrera Y., Paccagnella N., Castellani G., De Martin S., Ferlin M.G. (2018). Targeting RORs nuclear receptors by novel synthetic steroidal inverse agonists for autoimmune disorders. Bioorg. Med. Chem..

[133] Pastwińska J., Karaś K., Sałkowska A., Karwaciak I., Chałaśkiewicz K., Wojtczak B.A., Bachorz R.A., Ratajewski M. (2022). Identification of Corosolic and Oleanolic Acids as Molecules Antagonizing the Human RORγT Nuclear Receptor Using the Calculated Fingerprints of the Molecular Similarity. Int. J. Mol. Sci..

[134] Solt L.A., Burris T.P. (2012). Action of RORs and Their Ligands in (Patho)physiology. Trends Endocrinol. Metab..

[135] Zou H., Yang N., Zhang X., Chen H.-W. (2022). RORγ is a context-specific master regulator of cholesterol biosynthesis and an emerging therapeutic target in cancer and autoimmune diseases. Biochem. Pharmacol..

[136] Zou H., Zou H., Yang Y., Yang Y., Shi Z., Shi Z., Wu X., Wu X., Liu R., Liu R. (2022). Nuclear receptor RORγ inverse agonists/antagonists display tissue- and gene-context selectivity through distinct activities in altering chromatin accessibility and master regulator SREBP2 occupancy. Pharmacol. Res..

[137] Wang Y., Huang Z., Chen C.Z., Liu C., Evans C.P., Gao A.C., Zhou F., Chen H.-W. (2020). Therapeutic Targeting of MDR1 Expression by RORγ Antagonists Resensitizes Cross-Resistant CRPC to Taxane via Coordinated Induction of Cell Death Programs. Mol. Cancer Ther..

[138] Kojetin D.J., Burris T.P. (2014). REV-ERB and ROR nuclear receptors as drug targets. Nat. Rev. Drug Discov..

[139] Zhou X., Chen H., Wei F., Zhao Q., Su Q., Lei Y., Yin M., Tian X., Liu Z., Yu B. (2020). The Inhibitory Effects of Pentacyclic Triterpenes from Loquat Leaf against Th17 Differentiation. Immunol. Investig..

[140] Tian X., Tang L., Wei F., Chen H., Sheng L., Yang Y., Zhou X., Li Y., Xu X., Zhang B. (2021). Pentacyclic triterpene compounds from loquat leaves reduce skin inflammation and epidermal hyperplasia in psoriasis via inhibiting the Th17 cells. Mol. Immunol..

[141] Fujita-Sato S., Ito S., Isobe T., Ohyama T., Wakabayashi K., Morishita K., Ando O., Isono F. (2011). Structural Basis of Digoxin That Antagonizes RORγt Receptor Activity and Suppresses Th17 Cell Differentiation and Interleukin (IL)-17 Production. J. Biol. Chem..

[142] Lamers C., Schubert-Zsilavecz M., Merk D. (2014). Medicinal chemistry and pharmacological effects of Farnesoid X Receptor (FXR) antagonists. Curr. Top. Med. Chem..

[143] Sun L., Cai J., Gonzalez F.J. (2021). The role of farnesoid X receptor in metabolic diseases, and gastrointestinal and liver cancer. Nat. Rev. Gastroenterol. Hepatol..

[144] Jiang L., Zhang H., Xiao D., Wei H., Chen Y. (2021). Farnesoid X receptor (FXR): Structures and ligands. Comput. Struct. Biotechnol. J..

[145] Makishima M., Okamoto A.Y., Repa J.J., Tu H., Learned R.M., Luk A., Hull M.V., Lustig K.D., Mangelsdorf D.J., Shan B. (1999). Identification of a nuclear receptor for bile acids. Science.

[146] Parks D.J., Blanchard S.G., Bledsoe R.K., Chandra G., Consler T.G., Kliewer S.A., Stimmel J.B., Willson T.M., Zavacki A.M., Moore D.D. (1999). Bile acids: Natural ligands for an orphan nuclear receptor. Science.

[147] Zhang Y., Lee F.Y., Barrera G., Lee H., Vales C., Gonzalez F.J., Willson T.M., Edwards P.A. (2006). Activation of the nuclear receptor FXR improves hyperglycemia and hyperlipidemia in diabetic mice. Proc. Natl. Acad. Sci. USA.

[148] Pellicciari R., Costantino G., Fiorucci S. (2005). Farnesoid X receptor: From structure to potential clinical applications. J. Med. Chem..

[149] Shi X., Chen Y., Zhou T., Wang J., Xu X., Chen L., Hu L., Shen X. (2018). HS218 as an FXR antagonist suppresses gluconeogenesis by inhibiting FXR binding to PGC-1α promoter. Metabolism.

[150] Festa C., Finamore C., Marchianò S., Di Leva F.S., Carino A., Monti M.C., del Gaudio F., Ceccacci S., Limongelli V., Zampella A. (2019). Investigation around the Oxadiazole Core in the Discovery of a New Chemotype of Potent and Selective FXR Antagonists. ACS Med. Chem. Lett..

[151] Mudaliar S., Henry R.R., Sanyal A.J., Morrow L., Marschall H., Kipnes M., Adorini L., Sciacca C.I., Clopton P., Castelloe E. (2013). Efficacy and safety of the farnesoid X receptor agonist obeticholic acid in patients with type 2 diabetes and nonalcoholic fatty liver disease. Gastroenterology.

[152] Sun Y., Zhang W., Li N., Guo S., Gao L., Ge N. (2023). Effect of Ursolic Acid Extracted from *Hippophae rhamnoides* L. on FXR Signaling Pathway in Liver of Rats with Alcoholic Liver Injury. Sci. Technol. Food Ind..

